# Extreme intra-familial variability of congenital central hypoventilation syndrome: a case series

**DOI:** 10.1186/1752-1947-7-117

**Published:** 2013-04-26

**Authors:** Elizabeth Bygarski, Melanie Paterson, Edmond G Lemire

**Affiliations:** 1 Department of Biology, John R. Brodie Science Centre, Brandon University, 270 18th Street, Brandon, MB R7A 6A9, Canada; 2 Division of Medical Genetics, Royal University Hospital, 103 Hospital Drive, Saskatoon, SK S7N 0W8, Canada; 3 Department of Pediatrics, University of Saskatchewan, 103 Hospital Drive, Saskatoon, SK S7N 0W8, Canada; 4 Department of Biology, University of Saskatchewan, W.P. Thompson Building, 112 Science Place, Saskatoon, SK S7N 5E2, Canada

## Abstract

**Introduction:**

Congenital central hypoventilation syndrome is an autosomal dominant disorder that classically presents as sudden death in infancy secondary to central hypoventilation. Most cases are caused by polyalanine repeat mutations in the paired-like homeobox 2B gene, *PHOX2B*. More severe disease is typically associated with nonpolyalanine repeat mutations. We report the case of a family with nonpolyalanine repeat mutations that uncharacteristically has many individuals who were mildly symptomatic and only diagnosed after genetic testing. We highlight the highly variable clinical presentation of this condition and the need for clinicians to remain vigilant.

**Case presentation:**

We identified 10 individuals in a large extended Caucasian family of German and Austrian background with congenital central hypoventilation syndrome.

Case 1: A 16-year old male proband presented for reproductive counseling. He had a previous history of apneic spells and Hirschsprung disease in the neonatal period. A *PHOX2B* nonpolyalanine repeat mutation was identified in the proband and used to screen his extended family.

Cases 2 to 10: Several mildly symptomatic family members (males aged 5, 13, 42 and 80 years; females aged 28, 44, 46 and 48 years) spanning four generations were identified after genetic screening. A newborn boy from this family was also recently diagnosed with Hirschsprung disease and went on to have an abnormal sleep study.

**Conclusions:**

In this report, we highlight the significant phenotypic variability of congenital central hypoventilation syndrome, previously thought to be a rare genetic condition. Given the extreme clinical variability, it is possible that the prevalence of congenital central hypoventilation syndrome in the general population is much higher than previous estimates. This is of major importance to all clinicians who will need to maintain a high index of suspicion for this not so rare and highly clinically variable genetic condition that spans all ages. As the familial mutation has been identified, presymptomatic and prenatal diagnostic testing are available options for family members.

## Introduction

Congenital central hypoventilation syndrome (CCHS), an autosomal dominant disorder that classically presents as sudden death in infancy secondary to central hypoventilation, was first reported in the 1970s [1-4]. The molecular basis of CCHS was identified in 2009 and attributed to mutations in the paired-like homeobox 2B gene, *PHOX2B*. Two types of mutations have been described: polyalanine repeat mutations (PARMs), which are frequent, have a variable clinical presentation, and disease severity correlates with allele size; and nonpolyalanine repeat mutations (NPARMS) that are usually associated with a more severe presentation including Hirschsprung disease (HSCR) and an increased tumor risk [1,3]. Milder forms of CCHS have been also described and are due to smaller PARMs [1-6].

We report on a family in which the 16-year old male proband presented with a previous history of HSCR and apnea (also known as Haddad syndrome) in the neonatal period. Genetic testing identified a *PHOX2B* NPARM, which is typical for severe CCHS. Many adult family members were only identified after genetic screening and found to be mildly symptomatic. In this report, we highlight the extreme clinical variability of CCHS and the need for all clinicians to remain vigilant.

## Case presentation

### Case 1 (III-3)

**Figure 1:**
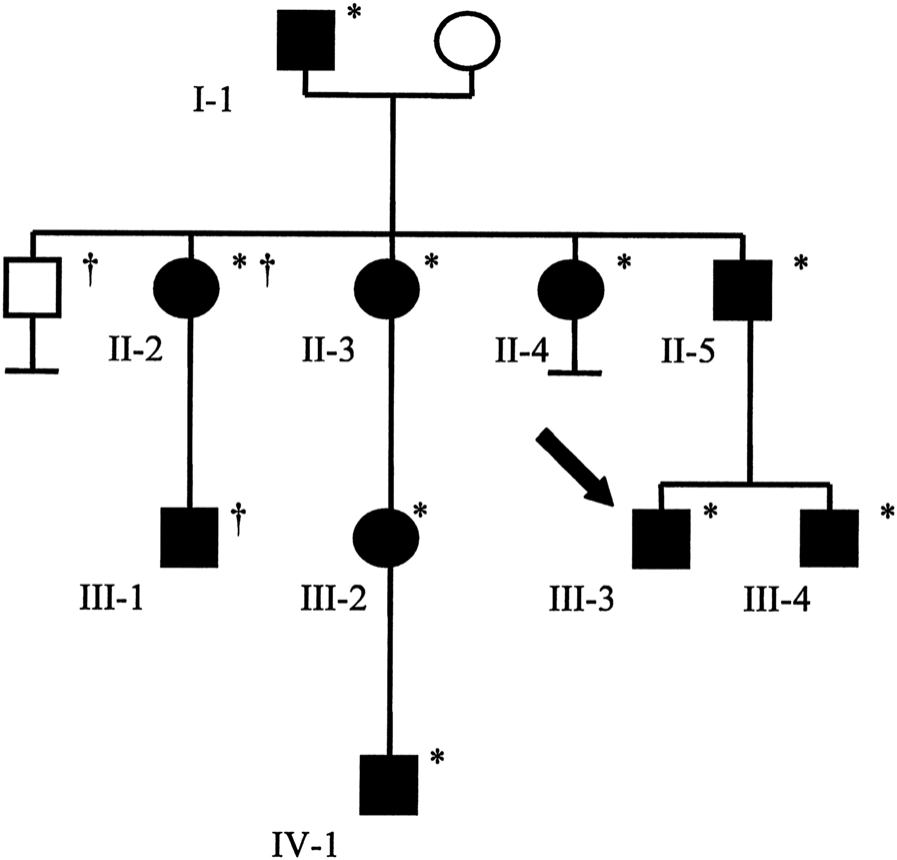
**Partial pedigree of the family with congenital central hypoventilation syndrome.** Squares indicate males and circles indicate females. Filled symbols identify individuals with confirmed and/or reported signs and symptoms of congenital central hypoventilation syndrome. The proband is indicated by the arrow. Confirmed *PHOX2B* mutation carriers are identified by an asterisk. A dagger denotes that the individual was not assessed.

The proband (III-3), a 16-year-old Caucasian adolescent of German and Austrian background, was referred for reproductive counseling for HSCR (Figure 1). He was born at term and transferred to a neonatal intensive care unit because of hyperbilirubinemia, where he developed central apneic spells with bradycardia and desaturations. He was eventually discharged home on caffeine. At age three weeks, he was readmitted because of abdominal distension, vomiting and dehydration. Radiographic findings were consistent with HSCR. He underwent a partial colectomy and colostomy during this admission followed by an endorectal pull-through at age 12 months. He was hospitalized three times by eight years of age for recurrent episodes of abdominal pain and distension. He has otherwise been healthy and his physical examination was unremarkable. Mutation analysis found no mutations in the *RET* gene. Targeted mutation analysis did not detect a polyalanine repeat expansion in the *PHOX2B* gene; however a novel frameshift mutation in exon 2 (c.391delC) associated with CCHS was identified. Our patient was referred to Cardiology, Ophthalmology and Respirology for assessment and management. His cardiac assessment was normal; there were minor pupillary abnormalities that did not require intervention. He had a normal pulmonary function test and was scheduled for an overnight sleep study, but failed to attend.

### Case 2 (II-5)

The proband’s 42-year-old father (II-5) was found to carry the identical *PHOX2B* mutation. A review of his medical history confirmed that he had unexplained episodes of diaphoresis and pupils that dilated poorly with atropine. A respiratory assessment found him to have a low resting oxygen saturation level (91%) and mild obstructive sleep apnea requiring nocturnal continuous positive airway pressure (CPAP).

### Case 3 (III-4)

The proband’s healthy 13-year-old brother had a remarkable neonatal history as a term baby with an admission to a neonatal intensive care unit for stridulous respirations and desaturations (O_2 _levels down to 60% to 70%) requiring supplementary oxygen. He was managed for possible necrotizing enterocolitis, a rare entity in term infants, with HSCR also under consideration in the differential diagnosis.

### Case 4 (I-1)

The proband’s 80-year-old paternal grandfather was on CPAP for severe obstructive sleep apnea and had had a cardiac arrest in 2007. He had a reported history of daily laxative use for chronic constipation and sluggish pupillary responses to atropine.

### Case 5 (II-4)

A 44-year-old paternal aunt had a childhood history of recurrent bronchitis and photophobia. Interestingly, she was found to have unexplained low resting oxygen saturation levels during a hospital admission for a cholecystectomy at age 25. She is now on CPAP for severe obstructive sleep apnea and has shown signs of pulmonary hypertension.

### Case 6 (II-3)

A 46-year-old paternal aunt has small pupils that react sluggishly to light, sleep apnea and occasional episodes of diaphoresis.

### Case 7 (III-2)

The 28-year old daughter of II-3, a paternal first cousin to the proband (III-3), had a history of recurrent abdominal pains and chronic constipation as a child.

### Case 8 (IV-1)

The newborn son of the III-2 was recently diagnosed with HSCR and went on to have an abnormal sleep study. He is being managed by noninvasive ventilation and is undergoing screening for neural crest tumors.

### Case 9 (II-2)

A 48-year-old paternal aunt reportedly has tested positive for the familial mutation, but no clinical information is available.

### Case 10 (III-1)

The five-year-old son of II-2 is reportedly being investigated for possible HSCR. Family history and clinical findings of all family members are summarized in Figure 1 and Table 1, respectively.

**Table 1:** Tabulation of clinical manifestations of individual family members

	**Case**									
	**1**	**2**	**3**	**4**	**5**	**6**	**7**	**8**	**9**	**10**
	**III-3**	**II-5**	**III-4**	**I-1**	**II-4**	**II-3**	**III-2**	**IV-1**	**II-2**	**III-1**
Pupillary abnormalities	+	+	+	+	+	+	?	?	?	?
Obstructive sleep apnea	-	+	-	+	+	+	-	-	?	?
Neonatal apneas/desaturations	+	?	+	?	?	?	?	+	?	?
Low resting O_2_ saturation levels	-	+	-	-	+	-	-	+	?	?
Pulmonary hypertension	-	-	-	-	+	-	-	-	?	?
Cardiac arrest	-	-	-	+	-	-	-	-	?	?
Hirschsprung disease	+	-	-	-	-	-	-	+	?	?+
Constipation	-	-	+	+	-	-	+	-	?	?
Episodic diaphoresis	-	+	-	-	-	+	-	-	?	?
Neural crest tumors	-	-	-	-	-	-	-	-	?	?

Present (+), Absent (−), (?) Unknown or not formally assessed.

## Discussion

CCHS is a disease of the autonomic nervous system that is not as rare as previously thought, with an estimated incidence of one per 200,000 live births [1,3,7]. Classically, it presents as sudden death in infancy because of a failure of autonomic control of ventilation during sleep (Ondine curse) [1,2,5]. Infants with CCHS typically do not breathe spontaneously for the first few months of life, but can develop breathing patterns while awake over time. The hypoventilation associated with CCHS is more severe in non-rapid eye sleep, which contrasts with other respiratory disorders [8]. Untreated, this may result in pulmonary hypertension, cor pulmonale and central nervous system hypoxic damage [1,5]. Other clinical manifestations of CCHS as a result of generalized autonomic nervous system dysfunction include cardiac arrhythmias, orthostatic hypotension, episodes of profuse diaphoresis, pupillary abnormalities, severe constipation, HSCR and neural crest tumors [1]. CCHS is diagnosed in newborns as clinical symptoms develop in the absence of any neuromuscular, cardiovascular or respiratory system abnormalities [1,3,5].

Late-onset CCHS (LO-CCHS) is diagnosed in adults who present with signs of central alveolar hypoventilation. They are at risk for polycythemia and pulmonary hypertension. Most adults with LO-CCHS carry a smaller expansion in *PHOX2B* when compared with infants with classic CCHS, but NPARMs have also occasionally been reported [1,2,5,9].

HSCR is a developmental disorder of the enteric system that is characterized by aganglionosis in the distal colon causing bowel obstruction. It occurs in approximately one out of every 5000 live births [6]. It generally presents as a failure to pass meconium as a neonate or, later in childhood, as chronic constipation, abdominal distension and emesis. Although HSCR can arise as an isolated finding, it can also be an associated feature in 16% to 50% of cases of CCHS [1,4,6,7,10].

CCHS is an autosomal dominant disorder caused by mutations in the *PHOX2B* gene on chromosome 4 [1,3]. This gene was originally identified in mice with abnormalities of the autonomic nervous system. Most cases are associated with PARMs in exon three [1]. Normal individuals carry a 20-alanine repeat sequence, while those with classic CCHS have expansions in the +5 to +13 range. Studies have demonstrated a direct correlation between the repeat number and disease severity. Individuals with small expansions (25 repeats) have LO-CCHS and rarely require ventilation, while those with larger expansions (27 to 33 repeats) often require continuous ventilatory support [1,3-5]. Less common are the NPARMs (frameshift and nonsense mutations) in *PHOX2B*. These are commonly associated with a more severe clinical phenotype including the need for continuous ventilatory support, tumors of neural crest origin and HSCR [1]. As with many autosomal dominant conditions, CCHS demonstrates variable expressivity and reduced penetrance. The family in our case was unusual in that most family members were relatively mildly affected and only identified later in childhood or as adults after the diagnosis was made in the proband (III-3), despite carrying an NPARM that is usually associated with more severe disease. This again confirms the highly variable clinical presentation for CCHS and the importance of sleep studies in apparently asymptomatic family members [11]. Phenotypic differences previously described in monozygotic twins with CCHS suggest a role for non-genetic factors in clinical variability and disease severity [12]. Reduced penetrance and variable expressivity has also been described in LO-CCHS [9,13].

*PHOX2B* mutation carriers are at risk for chronic hypoventilation and arrhythmias and must be assessed and managed by specialists familiar with CCHS. The American Thoracic Society has issued a clinical policy statement on diagnosis and treatment of CCHS that is largely based on expert opinion [1]. The primary goal of treatment is to ensure optimal ventilation and oxygenation through the use of mechanical assisted ventilation. Most individuals require at least nocturnal ventilation and some may need continuous ventilator support to avoid the long-term complications of chronic hypoxemia. In newborns and children, expert opinion recommends positive pressure ventilation via tracheostomy. In all cases, close monitoring is necessary to maintain normal oxygen levels for various activities and to minimize deleterious effects on cognitive development. Sedatives and central nervous system depressants should be avoided as they can depress ventilatory drive and, in the absence of ventilatory support, can lead to death.

Individuals with CCHS can survive to adulthood with appropriate management. The cause of death in individuals with CCHS relates to suboptimal ventilation, the use of substances that depress ventilatory control and cardiac arrhythmias. Screening recommendations for individuals with CCHS based on genotype are published [1]. Presymptomatic and prenatal diagnostic testing are available in instances where the disease-causing *PHOX2B* mutation has been identified.

## Conclusions

In this report, we highlight the importance of considering CCHS in the differential diagnosis for HSCR or when the medical or family histories are suggestive of generalized autonomic nervous system dysregulation. The clinical variability demonstrated by this family suggests that CCHS is likely an unrecognized entity and that previous frequency measurements are probably underestimates. All clinicians must maintain a high index of suspicion for CCHS in order to diagnose and manage mildly symptomatic cases, to avoid the associated long-term morbidity and mortality, and to permit genetic testing and counseling for families. Multidisciplinary involvement is necessary for appropriate treatment, management and monitoring of individuals with CCHS.

## Consent

Written informed consents were obtained from the patients and from the minors’ next of kin for publication of this manuscript and accompanying images. A copy of the written consents are available for review by the Editor-in-Chief of this journal.

## Competing interests

The authors declare that they have no competing interests.

## Authors’ contributions

EB reviewed the patient records, tabulated the clinical information and wrote the initial manuscript, which was submitted as part of an undergraduate student project. MP met with the family, provided genetic counseling and obtained patient consents. EGL supervised EB for her course work, examined the patients and modified the manuscript for publication. All authors read and approved the final manuscript.
